# Biomolecules resveratrol + coenzyme Q10 recover the cell state of human mesenchymal stem cells after 1-methyl-4-phenylpyridinium-induced damage and improve proliferation and neural differentiation

**DOI:** 10.3389/fnins.2022.929590

**Published:** 2022-08-31

**Authors:** Oscar R. Hernández-Pérez, Karen J. Juárez-Navarro, Nestor F. Diaz, Eduardo Padilla-Camberos, Miguel J. Beltran-Garcia, Dalila Cardenas-Castrejon, Héctor Corona-Perez, Claudia Hernández-Jiménez, Néstor E. Díaz-Martínez

**Affiliations:** ^1^Laboratorio de Reprogramación Celular y Bioingeniería de Tejidos, Biotecnología Médica y Farmacéutica, CONACYT Centro de Investigación y Asistencia en Tecnología y Diseño del Estado de Jalisco (CIATEJ), Guadalajara, Mexico; ^2^Instituto Nacional de Perinatología (INPER), Mexico City, Mexico; ^3^Biotecnología Médica y Farmacéutica, CONACYT Centro de Investigación y Asistencia en Tecnología y Diseño del Estado de Jalisco (CIATEJ), Guadalajara, Mexico; ^4^Departamento de Biotecnológicas y Ambientales, Universidad Autónoma de Guadalajara, Zapopan, Mexico; ^5^Foundation LCells, Guadalajara, Mexico; ^6^Cirugía Experimental, Instituto Nacional de Enfermedades Respiratorias (INER), Mexico City, Mexico

**Keywords:** mesenchymal stem cells, antioxidants, proliferation, resveratrol (3,5,4′-trihydroxy-trans-stilbene), ubiquinone (coenzyme Q10)

## Abstract

Neurodegenerative disorders are a critical affection with a high incidence around the world. Currently, there are no effective treatments to solve this problem. However, the application of mesenchymal stem cells (MSCs) and antioxidants in neurodegenerative diseases has shown to be a promising tool due to their multiple therapeutic effects. This work aimed to evaluate the effects of a combination of resveratrol (RSV) and coenzyme Q10 (CoQ10) on the proliferation and differentiation of MSC and the protector effects in induced damage. To characterize the MSCs, we performed flow cytometry, protocols of cellular differentiation, and immunocytochemistry analysis. The impact of RSV + CoQ10 in proliferation was evaluated by supplementing 2.5 and 10 μM of RSV + CoQ10 in a cellular kinetic for 14 days. Cell viability and lactate dehydrogenase levels (LDH) were also analyzed. The protective effect of RSV + CoQ10 was assessed by supplementing the treatment to damaged MSCs by 1-methyl-4-phenylpyridinium (MPP+); cellular viability, LDH, and reactive oxygen species (ROS) were evaluated.. MSCs expressed the surface markers CD44, CD73, CD90, and CD105 and showed multipotential ability. The combination of RSV + CoQ10 increased the proliferation potential and cell viability and decreased LDH levels. In addition, it reverted the effect of MPP+-induced damage in MSCs to enhance cell viability and decrease LDH and ROS. Finally, RSV + CoQ10 promoted the differentiation of neural progenitors. The combination of RSV + CoQ10 represents a potential treatment to improve MSCs capacities and protect against neurodegenerative damage.

## Introduction

Mesenchymal stem cells (MSCs) are multipotent cells with multiple differentiation capacities, which are considered a prime cell source to generate *in vitro* models of physiologic systems (Horwitz et al., 2005; Dominici et al., 2006; Juárez-Navarro et al., 2020). Furthermore, MSCs are a salient tool for studying pathologies to recreate models of chronic degenerative diseases (e.g., neural, musculoskeletal, cardiovascular, pulmonary, and autoimmune systems) (Yao et al., 2020). Recently, several trials employed human MSCs in the therapy of degenerative diseases (e.g., Alzheimer’s, Huntington’s, and Parkinson’s) (Afflerbach et al., 2020).

Degenerative diseases result from mitochondrial dysfunctions triggered by abnormalities in energy metabolism and increased cellular oxidative stress (Dias et al., 2013; Wang et al., 2019). Moreover, recent evidence suggests that oxidative stress is closely related to the normal cycle of MSCs as pathways in adhesion, proliferation, differentiation, cellular senescence, and aging (Kanda et al., 2011; Atashi et al., 2015). In line with this, the levels of reactive oxygen species (ROS) are associated highly with the regulation and deregulation of these pathways (Mody et al., 2001; Zhang et al., 2013).

Recent studies have shown the therapeutic potential of antioxidant molecules by repairing mitochondrial damage and reducing levels of abnormal oxidative stress in the cell (Ramana et al., 2018; Forman and Zhang, 2021). Resveratrol (RSV) and coenzyme Q10 (CoQ10) are antioxidant molecules with therapeutic potential. RSV has shown that antioxidant capacity positively affects cell senescence, increases cell viability, promotes differentiation, and enhances neural differentiation (Dai et al., 2007; Hu and Li, 2019; Songsaad et al., 2020). Previous studies also suggest RSV exerts neuroprotective effects and can scavenge ROS excess (Zhou et al., 2021). CoQ10 has been described to reduce oxidative stress, suppress ROS production, prevent apoptosis, and improve mitochondrial function (Quinzii and Hirano, 2010; Cornelius et al., 2017; Yousef et al., 2019). However, the possible implication of CoQ10 on MSCs differentiation has not been registered yet (Zhang et al., 2015; Huo et al., 2018).

To further explore the antioxidant effects of the RSV and CoQ10, we characterized a human mesenchymal stem cell (hMSC) line evaluating the role of these molecules on cell proliferation by performing a kinetic growth curve. Then, we studied the effect of these treatments on oxidative stress and cell viability by assessing 3-(4,5-dimethyl-2-thiazolyl)-2,5-diphenyltetrazolium bromide (MTT), LDH, and ROS levels. Subsequently, we realized stereological counts of cells immunostained for neural markers to register differentiation efficiency. Finally, the neuroprotective effect of RSV + CoQ10 was assessed by supplementing them after a damage induction with MPP+. The results obtained from this study are promising as we found significant changes in proliferation rates, differentiation efficiency, and cell viability of treated cells.

## Materials and methods

### Cell culture and reagents

Dulbecco’s Modified Eagle Medium (DMEM), trypsin, fetal bovine serum (FBS), and penicillin–streptomycin were purchased from Thermo Fisher Scientific. Dimethyl sulfoxide (DMSO), 3-(4,5-dimethylthiazol-2-yl)-2,5-diphenyltetrazolium bromide (MTT), coenzyme Q10 (CoQ10, Cat. C9438), and resveratrol (RSV, Cat. R5010) were acquired from Sigma-Aldrich company. Placenta-derived hMSCs were obtained from LCells foundation (Guadalajara, Jalisco, México; COFEPRIS 12-TR-14-039-0001). Cells were cultured on T125 culture flasks in DMEM supplemented with 10% FBS and 1% penicillin–streptomycin and incubated at standard conditions (37°C and 5% CO_2_). Cultures of T125 flasks were maintained until there was a 90–100% confluency for further experiments.

### Mesenchymal stem cell characterization

#### Flow cytometric analysis

We performed flow cytometry analysis to identify the presence of characteristic cell surface markers for hMSCs (CD44, CD73, CD90, and CD105) and the absence of hematopoietic markers (CD11b, CD19, CD45, and HLA-DRP). Cultured cells from T125 flasks were stained according to the manufacturer’s instructions (BD Biosciences, United States, 562245). After being stained, cells were analyzed on a BD FACSMelody flow cytometer system.

#### Three lineage differentiation *in vitro*

Multipotency of hMSCs was assessed by inducing the differentiation toward adipogenic, osteogenic, and chondrogenic lineages. We performed differentiation protocols by culturing the cells for 30 days in differentiation mediums: Human Adipocyte Differentiation Medium (Sigma 811D), Human Osteoblast Differentiation Medium (Sigma 417D), and Chondrocyte Differentiation Medium (Sigma 411D).

##### Adipocyte staining

Adipocyte fat vacuoles were stained using Oil Red O. Cells were washed twice with PBS, fixed with 4% paraformaldehyde, and washed once more. Then, 500 μl of 60% isopropanol was added and removed after 15 s, followed by 500 μl of Oil Red O for 10 min, and cells were washed with PBS.

##### Chondrocyte staining

Collagen fibers were stained with alcian blue. Cells were washed twice with PBS, fixed with 96% ethanol for 20 min, and washed once more, followed by the incubation with 1% acetic acid for 5 min, cells were washed with PBS, and alcian blue was added and incubated for 1 h.

##### Osteoblast staining

Osteoblast calcium deposits were stained with Von Kossa. Cells were washed twice with distilled water, fixed with 2 ml of cold 70% ethanol, and left overnight at −20°C. Ethanol was discarded, cells were washed once, and 500 μl of 5% AgNO_3_ was added; at last, staining was exposed to UV radiation at 120 μJ/cm^2^ for 5 min. All pictures were taken with a ZEISS Axio Vert. A1 microscope.

### Treatment preparation

Stock solutions of RSV, CoQ10, and RSV + CoQ10 were prepared by dissolving compounds in DMSO to achieve final stock concentrations of 2.5 and 10 mM for RSV and CoQ10, respectively. Treatment concentrations were 2.5, 10, and 2.5 + 10 μM, respectively. The dose range of RSV and CoQ10 was based on studies to exert antioxidant effects on MSCs (Zhang et al., 2015; Xinxin et al., 2016). Final DMSO concentrations did not exceed 0.1%, and control culture media were supplemented with DMSO to a final concentration of 0.1%.

### Kinetics cell proliferation assay

Cells were seeded at an initial density of 3 × 10^3^ in 24-well plates and cultured with the different treatments (RSV 2.5 μM, CoQ10 10 μM, and RSV + CoQ10). Cell proliferation was assessed by sensing cells from each treatment every 2 days until day 14. To sense, cells were detached and counted using Trypan Blue (1:1) exclusion in a Neubauer chamber. All experiments were performed in triplicates.

### Cell proliferation, damage induction, and differentiation toward neural lineage

We worked with four groups to evaluate the protective effect of RSV, CoQ10, and RSV + CoQ10 before or after damage induction and differentiation. Groups tested are as follows: Control (DMSO 0.1% added to proliferation and differentiation mediums); molecule proliferation (Mol. Prol.) for antioxidant molecule treatments (RSV + CoQ10) added during the proliferation stage; molecule differentiation (Mol. Diff.) for treatments supplemented just in differentiation stage into the culture medium; molecule both (Mol. Both) treatments added to both (proliferation and differentiation stages). Damage and no damage labels stand for cells damaged. Damage in MSCs was induced by cellular cytotoxicity to inhibit mitochondrial respiratory chain complex 1 using 1-methyl-4-phenylpyridinium (MPP+) (0.5 mM), a common neurotoxin used in models of Parkinson’s disease.

For 10 days, cells were seeded and cultured for the kinetics cell proliferation assay. We assessed the levels of LDH, ROS, and MTT at D2 and D10. On D10, cell damage was induced with 0.5 mM MPP+ in damage groups, according to Li et al. (2019). Then, differentiation was performed on D10 by changing the proliferation medium to the differentiation medium; DMEM/F12 with Glutamax supplemented with 2% of FBS, 0.2% of penicillin/streptomycin, 1% of N2 supplement, and EGF 10 ng/ml. Cells were cultured with this medium for 3 days; the medium was changed to DMEM/F12 with Glutamax supplemented with 2% of FBS, 0.2% of penicillin/streptomycin, 2% of B27 supplement, EGF 20 ng/ml, and bFGF 20 ng, and cells were cultured in this medium at standard conditions for 6 days. On D20, the differentiation medium was replaced for Neurobasal medium, FBS (10%), retinoic acid (1 μm), and 15 ng/ml of hBDNF for ten additional days medium change every 2 days (Portmann-Lanz et al., 2010; Kruminis-Kaszkiel et al., 2020). Finally, we assessed immunocytochemistry, LDH, ROS, and MTT levels posterior to the differentiation protocol (D31).

### Lactate dehydrogenase activity

Lactate dehydrogenase (LDH) activity was assessed with the LDH kit (Sigma MAK066) according to the manufacturer’s instructions. Test samples were prepared by taking 50 μl of culture medium, and all standards and test samples were prepared in triplicates in 96-well plates, to which 50 μl of the LDH master reaction mix was added and incubated for 2–3 min. LDH activity was evaluated at the beginning of the proliferation stage, before damage induction, and after differentiation and damage induction (days 1, 10, and 31, respectively). All assays were performed in triplicates.

### Reactive oxygen species

According to the manufacturer’s instructions, ROS production was assessed with the Fluorometric Intracellular ROS Kit (Sigma MAK144). We evaluated ROS in 24-well plates of seeded cells by adding 200 μl of Master Mix and incubating cells at standard conditions for 45 min. Fluorescence intensity (lex = 540/lem = 570 nm) was measured in a multi-well plate reader Bio-Rad xMARK, and all experiments were performed in triplicates.

### Cell viability

Cell viability was examined using the quantification of 3-(4,5-dimethyl-2-thiazolyl)-2,5-diphenyltetrazolium bromide (MTT) with a cell proliferation kit as described by the manufacturer’s instructions (Roche, 11465007001).

#### Immunocytochemistry

Cells were seeded at a density of 1 × 10^4^ cells/well in a chamber slide (Lab-Tek II, system 154534) previously coated with 0.1% gelatin. Forty-eight hours after, cells were washed twice with PBS and fixed with 4% paraformaldehyde solution for 20 min. Cells were washed with PBS/BSA solution (1:10) followed by permeabilization with 1X Triton X-100 and 10% Normal Calf Serum (NCS) solution. Cells were then incubated overnight at 4°C with the following primary antibodies: CD105 (Ab44967, 1:200), CD90 (Ab23894, 1:200), CD44 (Ab6124, 1:200), and β-Integrin (Ab52971, 1:200) for MSC characterization. Neural differentiation was assessed with the following antibodies: MAP2 (ab11267, 1:1000), SOX2 (ab5603, 1:500), GFAP (Sigma g3893, 1:500), β-tubulin (Merck MAB1637, 1:500), and Nestin (AB105389, 1:1000). Secondary antibodies used in both immunocytochemistry were Alexa 488 (Thermo Fisher A11034, 1:200) and Alexa 568 (Thermo Fisher A-11004, 1:200). Cell imaging was performed with a ZEISS Axio Vert. A1 microscope.

### Neural marker expression calculation

To determine the percent of differentiation toward neural cells, a counting of cell co-expressed signal for β-tub/SOX 2; GFAP/Nest or MAP2/SOX2 per five aleatory areas of 0.0288 mm^2^ each (area of a visual field: FOV through the 40× objective) and establishing a number relation representation (percent) concerning the total number of cell positives to DAPI.

### MALDI-TOF mass spectrometry lipid profile in mesenchymal and differentiated cells

Intact cells (1 μl of cellular suspension at 600 × 10^3^/50 μl) and chloroform extract samples at 1 and 4 μl were, respectively, spotted onto stainless steel MALDI target, dried at room temperature, and overlaid with 1 μl of 2,5 dihydroxybenzoic acid (DHB) matrix (30 mg/ml DHB dissolved in 30% methanol, 69% acetonitrile, and 1% trifluoroacetic acid). Samples were analyzed in triplicate. MS spectra were acquired using an Autoflex speed (Bruker Daltonics, Bremen, Germany) instrument equipped with a nitrogen laser at 355 nm. The spectra were acquired in reflectron mode using delayed extraction, either in positive or in negative ion mode, depending on the sensitivity, with a mass range of 200–1,200 Da. The acceleration voltage was 19 kV, and the reflectron voltage was 21 kV. The laser operated at a frequency of 1,000 Hz. Ion signals from 2,000 consecutive laser shots were summed into a spectrum. Data were acquired automatically and controlled by the Flex Analysis (version 3.4, Bruker Daltonics). The target *m/z* was selected after eliminating *m/z* peaks derived from DHB (matrix). The target *m/z* was compared with the database LIPID MAPS^®^ Lipidomics Gateway.^1^

Lipid extraction was achieved using cell pellets (10,000 cells) as follows: 1.1 ml isopropyl alcohol was added to cells and incubated for 30 min in a sonicator bath at 30°C. About 0.7 ml of chloroform was added and was sonicated again for 10 min. The suspension was centrifuged (10,000 × *g*, 10 min), and the lower chloroform phase was transferred to a glass vial and evaporated to dryness at 50°C. The sample was reconstituted in 300 μl chloroform. For MALDI analysis, 1 μl of DHB, 4 μl sample, and 1 μl of DHB were spotted on the stainless steel MALDI target.

### Statistical analysis

All experiments were performed in triplicates with primary cells from the same passage. The results are expressed as means ± SEM. Kolmogorov–Smirnov test was used to evaluate the normal distribution of the data. Differences between means were evaluated using one-way ANOVA followed when necessary (*P* < 0.05) by Dunnett’s test as a *post hoc* test. Significance in all tests was set at *P* < 0.05. Statistical parameters were computed using GraphPad Prism (Software, Version 9, San Diego, CA, United States).

## Results

### Characterization of human mesenchymal stem cells

**FIGURE 1:**
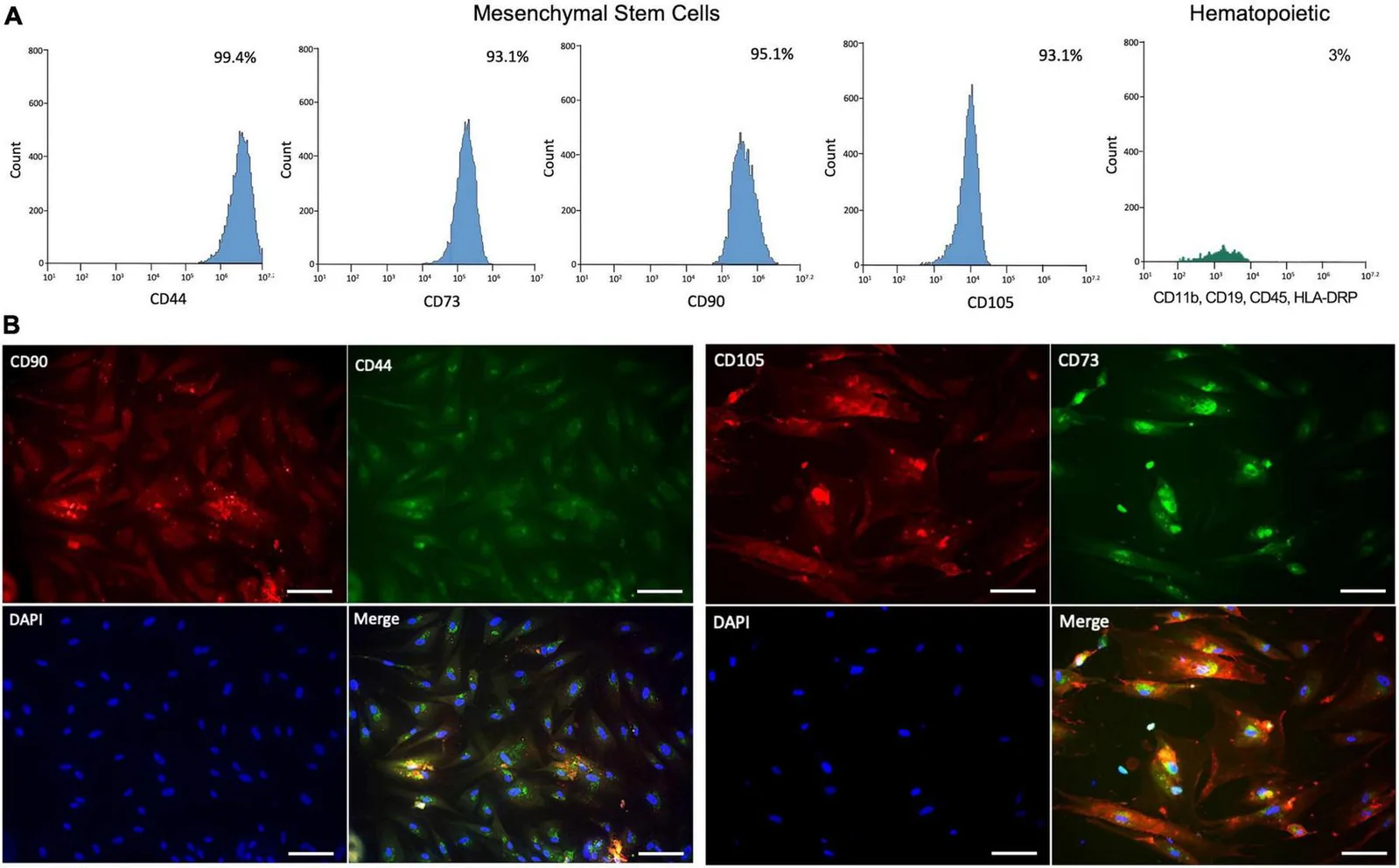
Characterization of human mesenchymal stem cells (hMSCs). **(A)** Analysis of surface expression markers expressed in percent of hMSCs (CD44, CD73, CD90, and CD105) and hematopoietic (CD11b, CD19, CD45, and HLA-DRP) by flow cytometry. **(B)** Immunocytochemistry profile of hMSCs expression of surface marker expression *in situ* under fluorescence microscopy. Scale bar: 25 μm.

**FIGURE 2:**
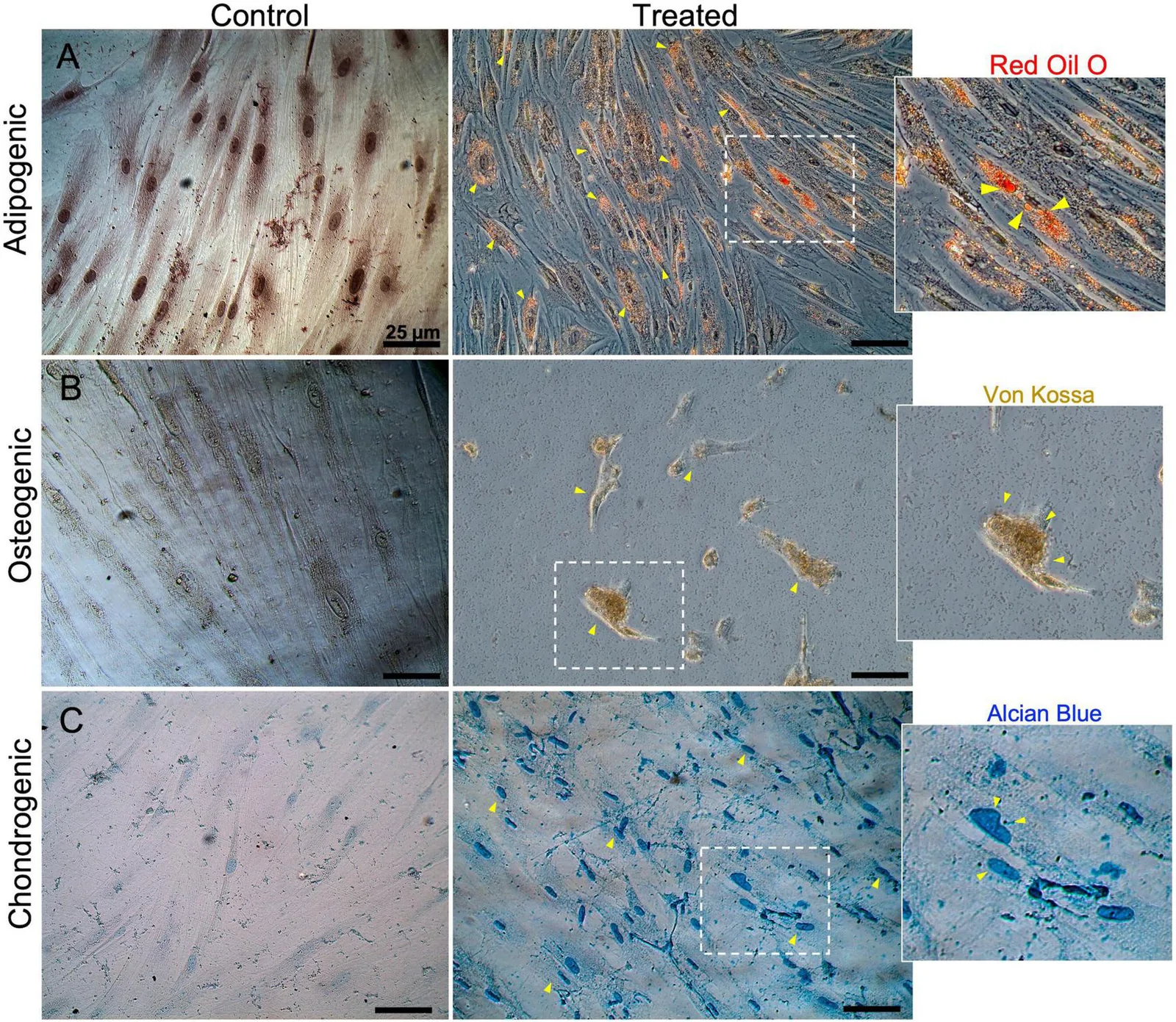
Characterization by multi-differentiation potential in human mesenchymal stem cells (hMSCs). hMSCs were differentiated into adipocytes, osteocytes, and chondrocytes. **(A)** Adipogenesis was confirmed by the presence of lipid droplets stained with Oil Red O. **(B)** Osteogenesis was confirmed with positive Von Kossa staining of osteoblast calcium deposits. **(C)** Chondrogenesis was indicated by the staining of collagen fibers with alcian blue.

To confirm the characteristics of MSCs in agreement with the international society for cells therapy (ISCT) (Dominici et al., 2006), we performed both an analysis of expression surface markers using flow cytometry and lineage differentiation protocols. We could confirm that hMSCs were positive to expression surface markers (>95%) for CD44, CD90, and less expression (93%) of both CD73 and CD105 (Figure 1A). The cocktail for the hematopoietic markers (CD11b, CD19, CD45, and HLA-DRP) was detected in only 3%. Furthermore, the immunocytochemistry profile of the hMSCs on the expression markers was possible to observe directly in cells (Figure 1B). Additionality was to verify the differentiation potential toward adipogenic, chondrogenic, and osteogenic lineages. We observed the presence of small adipocytic vesicles characteristic of the adipocytic cells (Figure 2A). The osteogenic lineage was corroborated due to the formation of extracellular calcium crystals (Figure 2B). Finally, were found secretions of cartilage-specific proteoglycans in the medium in the chondrogenic lineage (Figure 2C).

### A combination of resveratrol + coenzyme Q10 increases the proliferation and viability of human mesenchymal stem cells and reduces lactate dehydrogenase levels

**FIGURE 3:**
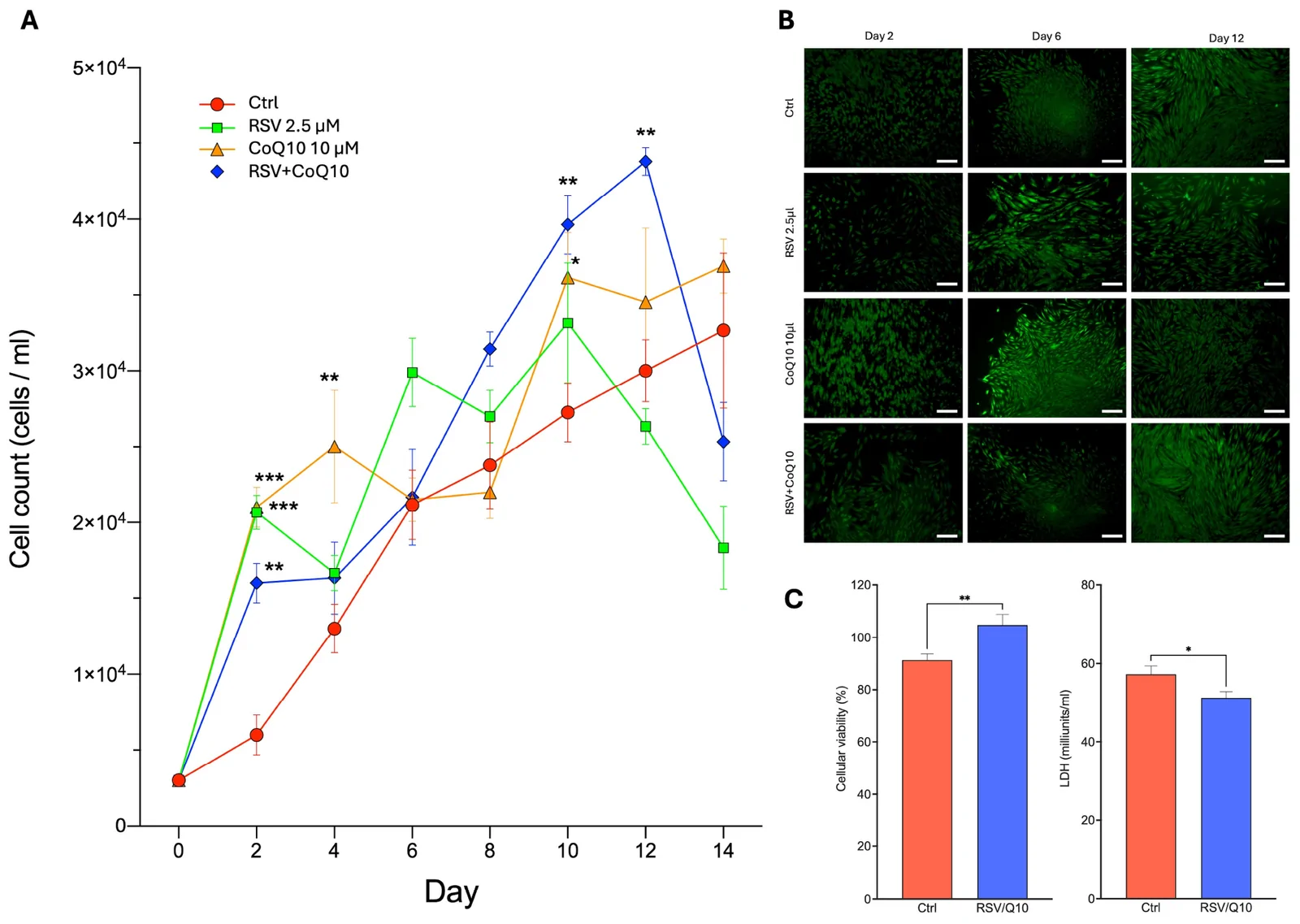
Effects of RSV + CoQ10 supplementation to human mesenchymal stem cells (hMSCs) on kinetic growth, cellular viability, and lactate dehydrogenase (LDH). **(A)** Applying RSV (2.5 μM), CoQ10 (10 μM), or combination to hMSCs increased the number of cells on day 2 vs. control. The RSV + CoQ10 combination significantly enhances the number of cells on days 10 and 12. **(B)** Representative pictures of confluency of hMSCs under fluorescence microscopy on three different days of kinetics. **(C)** On day 12, the application of RSV + CoQ10 increased cell viability. They reduced LDH levels compared to compare vs. control group. CoQ10, coenzyme Q10; RSV, resveratrol. *P < 0.05; **P < 0.01; ***P < 0.001. One-way ANOVA was followed by Dunnett’s test for kinetic growth curve and *t*-test for cell viability and LDH levels. Scale bar: 25 μm.

To evaluate the antioxidant effects on the proliferation of hMSCs, we performed a cellular kinetic supplement of 2.5 μM of RSV and 10 μM of CoQ10 individually and combined. hMSCs exhibited adequate proliferation potential during the kinetic. On day 2 was possible to observe a significantly enhanced number of hMSCs induced by RSV (2.07 ± 0.12 × 10^4^; *P* < 0.001), CoQ10 (2.10 ± 0.13 × 10^4^; *P* < 0.001), and combine (1.6 ± 0.13 × 10^4^; *P* < 0.01) vs. the control group (6 ± 1.3 × 10^3^). Furthermore, hMSCs supplement with RSV + CoQ10 showed a significant increase in the number of cells on days 10 (3.96 ± 0.19 × 10^4^; *P* < 0.01) and 12 (4.38 ± 0.09 × 10^4^; *P* < 0.01) to compare vs. the control group (2.72 ± 0.19 × 10^4^ and 3 ± 0.20 × 10^4^, respectively) (Figure 3A).

On day 12, the characteristic of hMSCs was to explore additional effects of RSV + CoQ10. As shown in Figure 3B, we analyzed the confluency of hMSCs of all treatments under phase-contrast microscopy on three different days (2, 6, and 12). We observed adequate morphological characteristics by the ISCT and an increase in the number of cells according to the kinetics. In addition, it was possible to keep a significant increase in the cell viability (Ctrl: 91.31 ± 2.31 vs. RSV + CoQ10: 104.8 ± 4.03; *P* < 0.01) and an opposite effect on LDH levels (Ctrl: 57.22 ± 2.14 vs. RSV + CoQ10: 51.22 ± 1.62; *P* < 0.05) (Figure 3C).

### Resveratrol + coenzyme Q10 maintains cellular viability and attenuates lactate dehydrogenase and reactive oxygen species production after induced cellular damage

**FIGURE 4:**
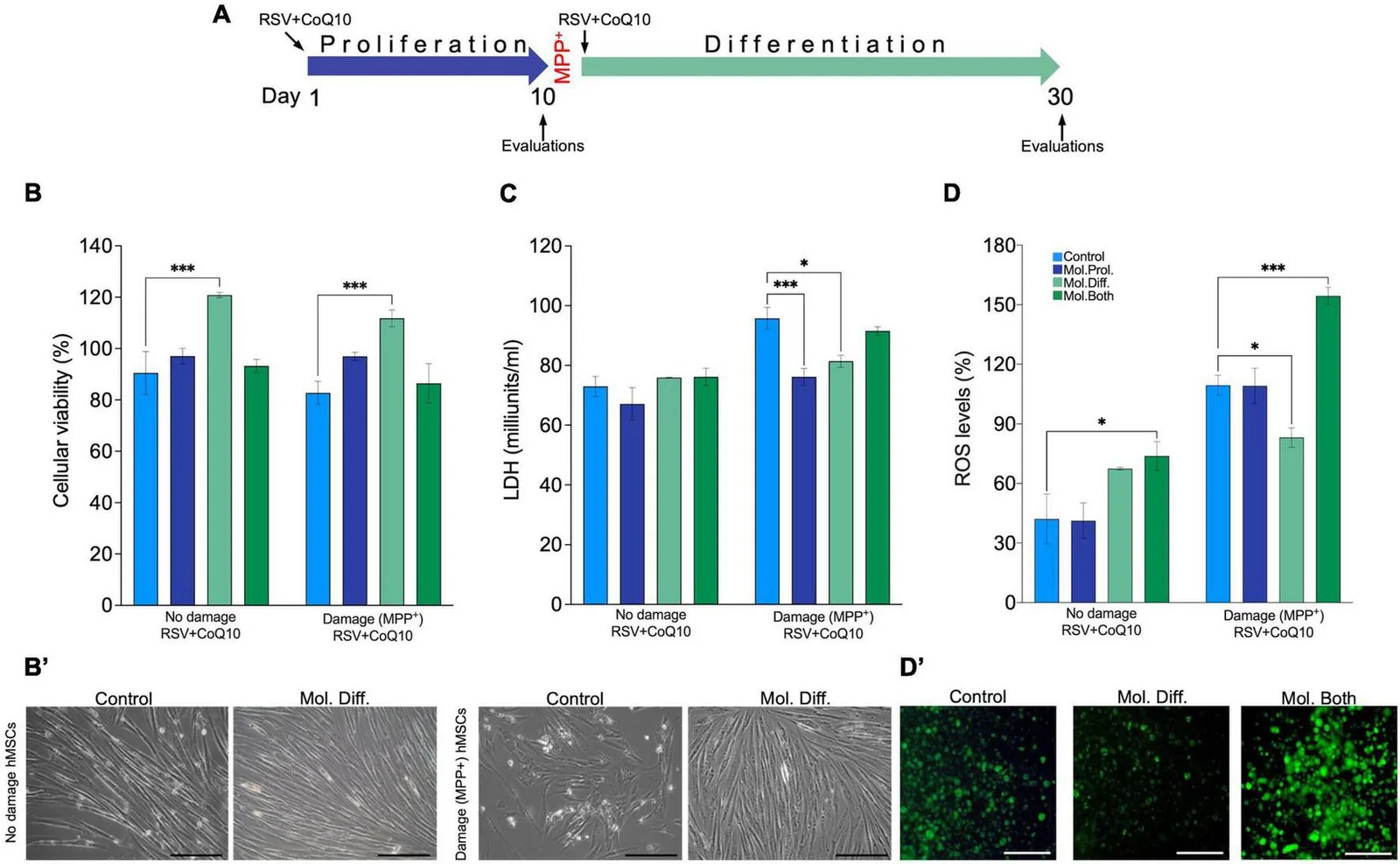
RSV + CoQ10 on human mesenchymal stem cell (hMSC) damage by MPP+, cellular viability, lactate dehydrogenase (LDH), and reactive oxygen species (ROS) evaluations. **(A)** Scheme representation of phases of the experiment, damage-induced time, and treatment application. **(B)** RSV + CoQ10 (2.5/10 μM) significantly enhances cell viability (%) during the differentiation stage in both no damage and damage hMSCs. **(B′)** Representative micrographs of cell viability. **(C)** Lactate dehydrogenase levels were increased in damaged hMSCs by MPP + and decreased posterior to the treatment application in proliferation and differentiation phases. **(D)** RSV + CoQ10 administration in both phases significantly increased ROS levels with no damage hMSCs. Finally, in hMSCs damage, the treatment increased significantly to apply in differentiation and both phases. **(D′)** Representative microphotography of fluorescence ROS assay. CoQ10, coenzyme Q10; RSV, resveratrol. ^∗^*P* < 0.05; ^∗∗∗^*P* < 0.001. One-way ANOVA was followed by Dunnett’s test. Scale bar: 100 μm.

To demonstrate the antioxidant effects of RSV + CoQ10 on MPP+-caused damage, we performed evaluations of cellular viability (MTT and LDH assay) and oxidative stress (ROS, extracellular production) at the end of the proliferation, differentiation, or the entire process (Figure 4A).

As shown in Figure 4B, the supplementation of RSV + CoQ10 in the differentiation stage increased the percent of cellular viability in both non-damaged (128.80 ± 4.45; *P* < 0.001) and damaged hMSCs induced by MPP+ (111.80 ± 3.23; *P* < 0.001) to compare with the control group (90.48 ± 8.37 and 82.74 ± 4.45, respectively). In Figure 4B’, we observe a representative cellular confluence and a viability increase. The extracellular levels of LDH in non-damaged hMSCs do not affect the application of RSV + CoQ10; however, with the supplementation of RSV + CoQ10 in damaged hMSCs, a reduction in LDH levels was observed. The best efficiency in the LDH reduction was to supplement the treatment in the proliferation stage (76.16 ± 2.81; *P* < 0.001); a discrete reduction was observed to apply the treatment in the differentiation stage (81.40 ± 2; *P* < 0.05) (Figure 4C).

The oxidative stress evaluations showed that all groups of non-damage hMSCs were minor to 90% of ROS, as shown in (Figure 4D’). Only in the group treated continuously (proliferation and differentiation) with RSV + CoQ10, it was possible to observe a significatively increase in the percent the ROS production (73.83 ± 7.27; *P* < 0.05) in comparison with the control group (42.08 ± 12.54). In the case of damaged hMSCs, the levels of ROS in all groups were about double that in non-damaged hMSCs. However, the application of the treatment produced two different effects, a greater ROS level when the treatment was applied continuously (154.50 ± 4.33; *P* < 0.001); in contrast, administering the treatment in the differentiation observed diminished ROS production (83.13 ± 4.89; *P* < 0.05) (Figure 4D).

### Supplementation of resveratrol + coenzyme Q10 to human mesenchymal stem cells increases cellular markers of neural stem cells posterior to damage-induced human mesenchymal stem cells

**FIGURE 5:**
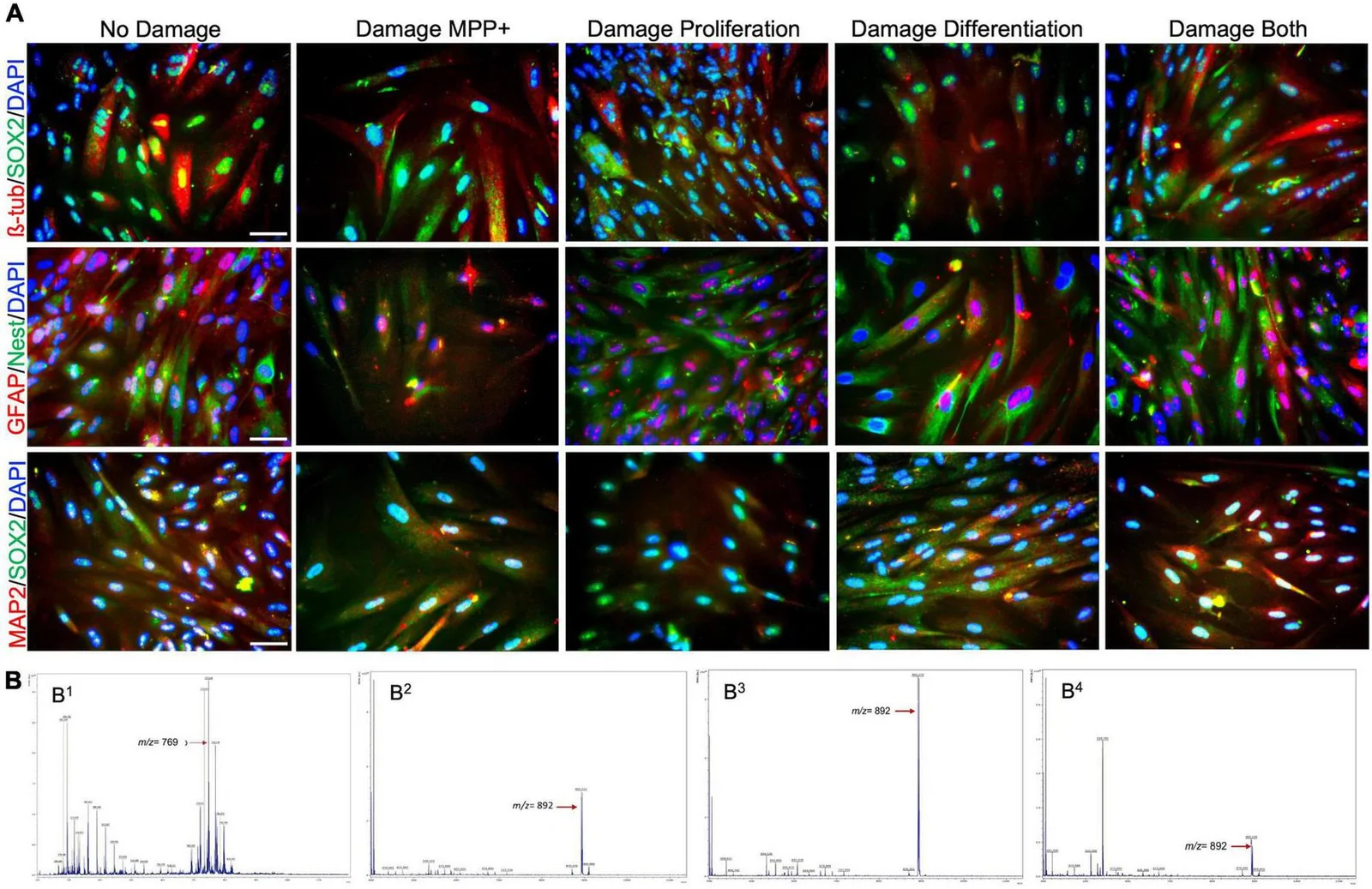
Expression of cell surface antigen markers of neuronal stem cells (NSCs) and MALDI-TOF spectra during cell differentiation. **(A)** Colocalization of antigens β-tubulin/SOX2 (red/green), GFAP/Nestin(red/green), and MAP2/SOX2 (red/green), and nuclear counterstaining marker DAPI (blue), in different conditions posterior to differentiation protocol from hMSCs to NSCs. **(B)** MALDI-TOF spectra of NSCs from human mesenchymal stem cells. (B^1^) hMSCs using DHB30, (B^2^) hMSCs differentiated using DHB30, (B^3^) hMSCs damage induced by MPP without antioxidants, and (B^4^) hMSCs damage induced by MPP with antioxidants during differentiation. Scale bar: 25 μm.

The use of MPP+ has been used as a degenerative-damage model. To explore the protective and differentiation effects of RSV + CoQ10, we administered the treatment in different stages and evaluated changes that occur during differentiation using MALDI-TOF and the expression of markers β-tubulin, SOX2, GFAP, Nestin, and MAP2 (Figure 5A).

**FIGURE 6:**
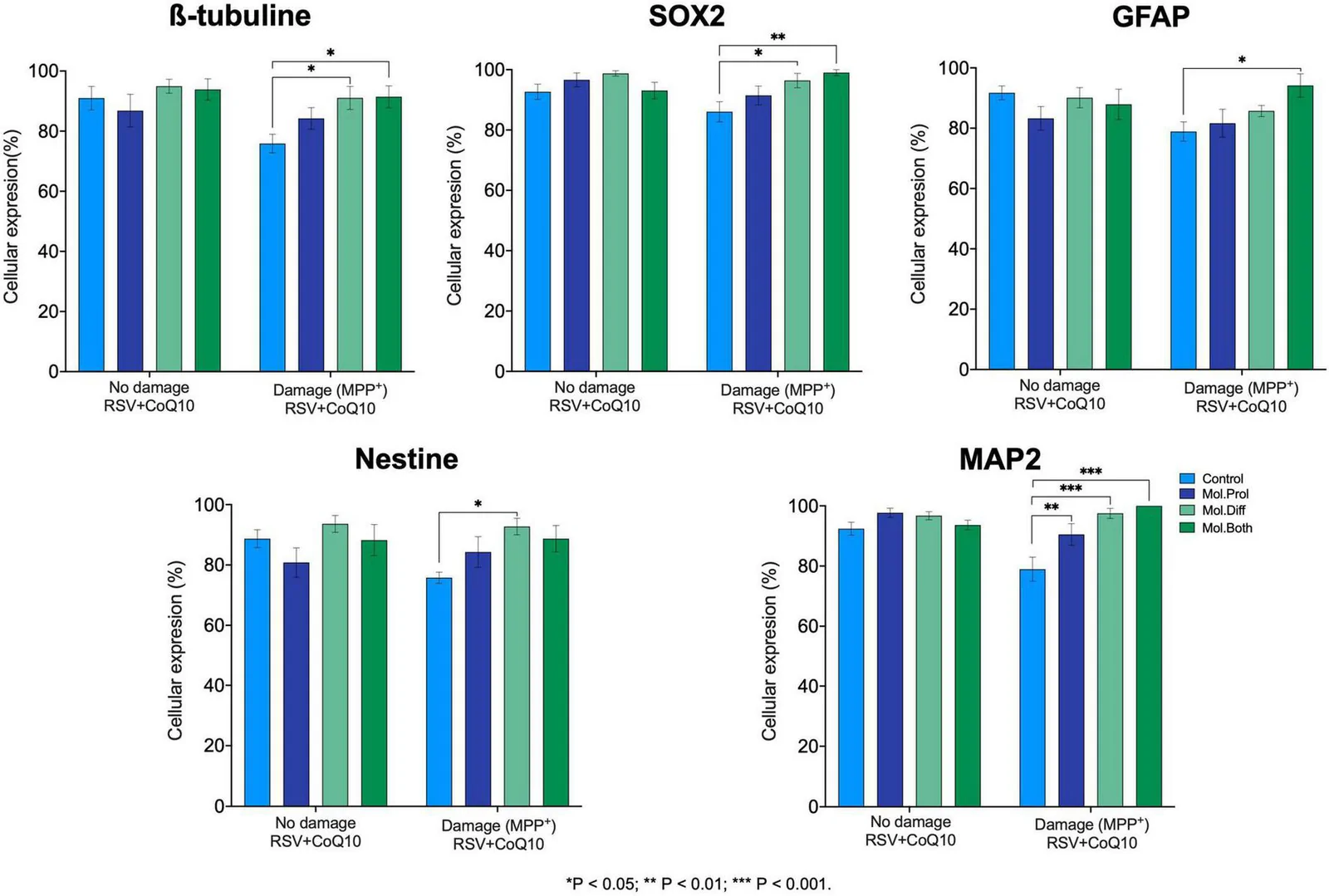
Cellular expression of neural stem cell (NSC) markers from human mesenchymal stem cells (hMSCs). The application of RSV + CoQ10 did not induce effects on the expression of neural markers when applied to hMSCs without damage; however, when supplemented after the induced damage, it showed a significant recovery effect on the cellular expression of all neural markers. CoQ10, coenzyme Q10; RSV, resveratrol. ^∗^*P* < 0.05; ^∗∗^*P* < 0.01; ^∗∗∗^*P* < 0.001. One-way ANOVA was followed by Dunnett’s test.

In no damage hMSCs, we do not observe differences in marker expression vs. controls (β-tubulin 90.97 ± 3.88; SOX2 92.66 ± 2.54; GFAP 91.71 ± 2.28; Nestin 88.72 ± 2.96, and MAP2 92.41 ± 2.14) to analyze the stereological counts. However, we found differences in hMSCs-induced damage on the expression of the markers after RSV + CoQ10 supplementation. It was only possible to observe an increase in MAP2 (*P* = 0.0021) when the treatment was a supplement in the proliferation phase. During the differentiation phase, the administration of antioxidants induced an increase in the expression of β-tubulin (*P* = 0.0141), SOX2 (*P* = 0.0124), Nestin (*P* = 0.0154), and MAP2 (*P* < 0.001). The application of RSV + CoQ10 in all development increased the expression of β-tubulin (*P* = 0.0116), SOX2 (*P* = 0.0014), GFAP (*P* = 0.0187), and MAP2 (*P* < 0.001) (Figure 6).

The lipid profiles for differentiated and undifferentiated hMSCs were evaluated using MALDI-TOF mass spectrometry. The positive ion MALDI-TOF mass spectrum for undifferentiated hMSCs compared to the matrix (DHB) spectrum allowed the identification of phospholipid (PL) molecular species. Figure 5B shows that peaks between *m/z* 650 to *m/z* 956 have different intensities in hMSCs. Those peaks show that they are mainly related to phosphatidylcholine (PC). The most abundant was PC (32:2) or PC [16:1/16:1 + H + K + ]+ at *m/z* 769.068 followed by PC (O-34:1) or PC [O-16:0/18:1 + H + ]+ at *m/z* 747.309, PC (34:3) [16:0/18:3 + K]+ at *m/z* 794.791, and PC (36:5) PC [18:1/18:4 + K]+ at *m/z* 818.516. The small peak at m/z 662.4 is determinative of vinylic cleavage of the C-C bond immediately distal to the C = C bond at n-9 in sn2 of PC phospholipid.

On the contrary, a significant change in the molecular profile of PCs was observed in differentiated cells or neural progenitors. The main peak was at *m/z* 892.168 PC(40:2), PC [20:0/22:2 + Na + ]+, followed by 870.260 [20:0/22:2 + H + ]+. Also, we found the disappearance of those PCs present in undifferentiated cell membranes. The PC at *m/z* 892.168 remains present in the differentiated cells but with slight variations (intensity) in the cells treated with MPP+. Differentiated cells also show peaks between 500 and 600 that suggest the presence of lysophosphatidylcholine (LPC), such as LPC 16:0, LPC 18:0, and LPC 18:1. The results here corroborate the abundance of PC in both mesenchymal and differentiated cells.

## Discussion

In this study, we provide evidence to demonstrate that supplementing the combination of RSV + CoQ10 to hMSCs increased proliferation, improved cellular viability, and reduced levels of LDH. Furthermore, we found that the application of RSV + CoQ10 during the differentiation phase to damage-induced hMSCs by MPP+ increased cellular viability (percent), reduced oxidative stress levels (LDH and ROS), and enhanced cellular differentiation to neural stem cells (NSCs). In agreement with this, phosphatidylcholine (a marker associated with cellular differentiation) was found in all treatments.

To verify the identity of hMSCs derived from the placenta used for the study, we performed the immunophenotypic analysis (Figures 1, 2). Following the proposed by ISCT (Dominici et al., 2006), typical cell membrane markers CD44, CD73, CD90, and CD105 (Beyer Nardi and da Silva Meirelles, 2006) were detected using flow cytometry (>90%) in hMSCs used in this study. Furthermore, a report has shown that CD73, CD90, and CD105 are general markers accepted for the expansion and isolation of hMSCs (Chamberlain et al., 2007; Ramos et al., 2016), and CD44 has been reported as a specific identity in human cells (Davoodian et al., 2014). In line with this, the presence of all markers was corroborated under microscopy analysis using the immunocytochemical method. In addition, the characteristic ability to differentiate to specific cell lineages was studied through the differentiation protocols, obtaining an adequate differentiation potential as described in previous work on managing human hMSCs, and by the ISCT (Heathman et al., 2016; Samsonraj et al., 2017). Therefore, based on the results exposed in the characterization, we can assure that the cells used in this work adequately meet the criteria established for hMSCs.

The result obtained on the kinetic growth showed normal behavior, an initial latency phase posterior to exponential growth, and in the end, a stationary phase in agreement with the typical behavior reported for normal hMSCs (Colter et al., 2001). It is important to note that in this work, the treatments are applied continuously for 14 days compared to previous studies; the application was with a maximum of 48 h or before the evaluations.

On day 14, a decrease in the number of hMSCs was observed. A possible explanation for this is the cumulative superoxide anion induced by RSV + CoQ10 posterior to 14 days of treatment, resulting in the hMSCs death. In support of this has been described that the oxidation of CoQ10 has been associated with ROS production, and supraphysiological levels result from a mitochondrial disruption of redox homeostasis, activating cell death (Linnane et al., 2007). In addition, the oxidation of both CoQ10 and RSV has been shown to reduce pancreatic (Dadali et al., 2021) and colon (San Hipólito-Luengo et al., 2017) cancer cells, respectively, through the activation of death pathways induced cytotoxicity and increase the percentage of apoptosis.

There is growing evidence that PLs are essential in measuring cell integrity, signal transduction, and differentiation in pluripotent stem cells. Current reports indicate that the phospholipid profile indicates cellular potency and makes cell lineages different (Prabutzki et al., 2020; Burk et al., 2021; Symons et al., 2021). Based on the low amount of cellular material (10,000 cells) used here for the identification of PL, we can suggest that PC (32:2) and PC (40:2) species are the majority of differentiated cells and neural progenitors. We found that adding antioxidant molecules decreases PC (40:2) at *m/z* 892.168 but might increase LPC molecules. The identification of phospholipids by MALDI-TOF may vary due to the diversity of formed adducts. In this work, we consider the formation of adducts with Na^+^ and K^+^ for all PC species according to LIPIDMAP.

The addition of RSV, CoQ10, or in combination with the hMSCs improved the number of cells during the growth curve, increased cell viability, and decreased levels of LDH. These effects are supported by what was previously described regarding the antioxidants effects of molecules by reducing oxidative stress and ROS (Zeng et al., 2015; Kwon and Park, 2020), thereby improving cell proliferative (activating ERK1/2 and p38 MAPK), survival, and differentiation capacity when supplemented in cell cultures (Dai et al., 2007; Shaban et al., 2017; Shafi et al., 2019).

Our results showed that the application of RSV + CoQ10 induced effects on cell viability, LDH, and ROS levels, to no damage and damage-induced hMSCs, and that effect depends on the phase in which treatment was applied. The mechanisms by which RSV + CoQ10 exerts its effects may occur at the mitochondrial level. Mitochondria play a principal role in oxidative phosphorylation, electron transport, maintaining intracellular calcium levels, and regulating pathways within the cell cycle. In support of this, RSV increases mitochondrial biogenesis and the activity of complex one in the respiratory chain and reduces oxidative stress activating the AMPK/sirtuin 1/peroxisome proliferator-activated receptor-PGC-1α pathway (Ferretta et al., 2014; Chen et al., 2017; Palle and Neerati, 2018). In addition, CoQ10 has been described as a cofactor in the electron transport chain, promoting ATP production and functioning as a powerful antioxidant by interacting with α-tocopherol to neutralize free radicals (Hernandez-Camacho et al., 2018; Manzar et al., 2020).

There is ample evidence of mitochondrial damage and dysfunction with neurodegenerative diseases (e.g., Alzheimer’s, Huntington’s, and Parkinson’s).

We performed a model of damage in hMSCs to apply MPP+ used in studies about neural degeneration (Jackson-Lewis and Smeyne, 2005), and posterior RSV + CoQ10 treatment observed a restoration and improvement in cell viability, which decrease both in LDH (in all groups treated) and ROS levels (except on Mol. Both group). The effect of RSV + CoQ10 enhanced cell viability on hMSCs obtained in the kinetic growth is corroborated to apply in hMSCs no damage during differentiation phase, increased the percentage of cells (%). Respect the effect on hMSCs damage induced; the treatment restored levels of viability (similarly to observed in no damage MSC) when treated in proliferation and both phases (30 days continuously); a dramatic increase was observed in viability to apply the treatment only on the differentiation phase.

As described above, the application of RSV + CoQ10 induced differential effects in hMSCs with and without damage, obtaining results in damaged hMSCs. The possible explanation may be that MPP+ affects the average mitochondrial level and the membrane, thereby inducing overproduction of ROS and a high release of LDH into the medium. There exists a correlation between the increase in the cell viability and a diminish of LDH and ROS levels posterior to applying RSV + CoQ10 during the differentiation phase. Previously, it has been demonstrated that LDH is an enzyme released in supernatant when de plasma membrane is damaged, and the high levels of LDH are related to dead cells (Caviedes-Bucheli et al., 2006). In cultures of MSC, the levels of ROS are commonly used to measure levels of oxidative stress and have been shown that high levels induce cellular death (Denu and Hematti, 2016).

We observed a reduction of ROS when administered treatment during the differentiation phase in damage hMSC. Per this, it has been described that antioxidants prevent and moderate ROS accumulation by inactivation of reactive species and facilitating the transcription factors (Nrf2) and expression of antioxidants enzymes (SOD1) (Dare et al., 2009; Gibellini et al., 2015). Nonetheless, it is possible to observe that previously detected positive effects were reversed when RSV + CoQ10 was administered during both phases. This result is attributable to the fact that it has been reported that oxidation of higher levels of RSV and CoQ10 elicited a dysfunctionality transference of electrons and improved mitochondrial electron escape as a result of enhanced ROS production (Zheng et al., 2018; Dadali et al., 2021). In addition, the excessive administration of antioxidant compounds can induce cell arrest, overproduction of ROS, and DNA damage, inducing premature senescence (Kornienko et al., 2019).

We observed a positive expression of markers β-tubulin, SOX2, GFAP, Nestin, and MAP2 posterior to the differentiation protocol. The appearance of these markers indicates that the differentiation process is completed since it has been shown in other works that the expressions of β-tubulin, Nestin, and MAP2 are characteristic markers of neuronal cells (Rafieemehr et al., 2015; Marei et al., 2018). In contrast, the expression of the protein GFAP is associated with grail cells (astroglia). Furthermore, in the case of SOX2, it is known to be a transcription factor necessary for the long-term self-renewal of neuronal stem cells (Pagin et al., 2021).

Resveratrol + coenzyme Q10 treatment did not influence the differentiation of hMSCs. However, in the hMSCs with damage, the markers were restored when applying RSV + CoQ10. In support of this, Jahan et al. (2018) reported restoring the impaired neural differentiation capacity of MSCs, induced by the neurotoxic organophosphate pesticide (Jahan et al., 2018a). The differentiation effects of RSV + CoQ10 may occur at the level of the signaling cascade, increasing the levels of protein kinase A, GSK-β, and ERK1/2 and thus an increase in CREB phosphorylation (Jahan et al., 2018b).

Resveratrol + coenzyme Q10 supplementation is a promising therapeutic alternative since, in support of our study, it has been reported that its application to MSC cultures increases cell proliferation, rescues mitochondrial functions and reduced oxidative stress, promotes neural differentiation, and increases dendritic growth (Schulz and Beal, 1995). In addition, clinical studies have shown that its administration preserves mitochondrial function by reducing neural loss and delaying functional decline in patients with neurodegenerative diseases, particularly Parkinson’s disease (Schulz and Beal, 1995; Yang et al., 2016). Finally, a continuously local administration of antioxidants or in combination with medical devices has shown a neuroprotective effect.

## Conclusion

The results of this work demonstrate that RSV + CoQ10 (2.5/10 μM) supplementation to hMSCs reverses damage in a neurodegeneration model using MPP+ by increasing cell viability, reducing oxidative stress levels, and restoring cell expression of neural markers. Furthermore, RSV + CoQ10 in growth kinetics increased the number of cells by improving cell viability and reducing LDH levels. These results allow us to consider the application of RSV + CoQ10 as a possible neuroprotective adjuvant with a future application in treating neurodegenerative diseases. To confirm the neuroprotective effect of RSV + CoQ10, it is necessary to provide antioxidant treatment in animal models of neurodegeneration induced by 6-OHDA to evaluate its protective effects on dopaminergic neurons *in vivo*.

## Data availability statement

The original contributions presented in this study are included in the article/supplementary material, further inquiries can be directed to the corresponding author.

## Author contributions

OH-P, KJ-N, and ND-M participated in the formulation or evolution of the general goals and objectives of the research and performed the analysis and interpretation of data and the creation and/or presentation of the published work, specifically writing the initial draft. OH-P, KJ-N, and MB-G carried out the research process and specifically the experiments, or data/evidence collection. OH-P, KJ-N, MB-G, ND, EP-C, DC-C, HC-P, and CH-J made substantial contributions to the preparation, creation, and/or presentation of the published work, specifically data visualization/presentation. ND-M participates in the preparation, creation and/or presentation of the work published by the original research group, specifically critical review, comment or review, including the pre- and post-publication stages. All authors discussed the results, revised the manuscript, and read and approved the final manuscript.

## Footnotes

1
http://www.lipidmaps.org

## Abbreviations

MTT: 3-(4,5-dimethyl-2-thiazolyl)-2,5-diphenyltetrazolium bromide
MPP+: v1-methyl-4-phenylpyridinium
CoQ10: coenzyme Q10
DMSO: dimethyl sulfoxide
DMEM: Dulbecco’s Modified Eagle Medium
FBS: fetal bovine serum
LDH: lactate dehydrogenase
MSCs: mesenchymal stem cell
ROS: reactive oxygen species
RSV: resveratrol.
