# Correction: Biomolecules resveratrol + coenzyme Q10 recover the cell state of human mesenchymal stem cells after 1-methyl-4-phenylpyridinium-induced damage and improve proliferation and neural differentiation

**DOI:** 10.3389/fnins.2026.1951334

**Published:** 2026-09-04

**Authors:** Oscar R. Hernández-Pérez, Karen J. Juárez-Navarro, Nestor F. Diaz, Eduardo Padilla-Camberos, Miguel J. Beltran-Garcia, Dalila Cardenas-Castrejon, Héctor Corona-Perez, Claudia Hernández-Jiménez, Néstor E. Díaz-Martínez

**Affiliations:** 1 Laboratorio de Reprogramación Celular y Bioingeniería de Tejidos, Biotecnología Médica y Farmacéutica, CONACYT Centro de Investigación y Asistencia en Tecnología y Diseño del Estado de Jalisco (CIATEJ), Guadalajara, Mexico; 2 Instituto Nacional de Perinatología (INPER), Mexico City, Mexico; 3 Biotecnología Médica y Farmacéutica, CONACYT Centro de Investigación y Asistencia en Tecnología y Diseño del Estado de Jalisco (CIATEJ), Guadalajara, Mexico; 4 Departamento de Biotecnológicas y Ambientales, Universidad Autónoma de Guadalajara, Zapopan, Mexico; 5 Foundation LCells, Guadalajara, Mexico; 6 Cirugía Experimental, Instituto Nacional de Enfermedades Respiratorias (INER), Mexico City, Mexico

**Keywords:** mesenchymal stem cells, antioxidants, proliferation, resveratrol (3, 5, 4′-trihydroxy-trans-stilbene), ubiquinone (coenzyme Q10)

There was a mistake in Figure 3 as published. Images from panel **(b)**, specifically the brightfield photographs, were incorrectly duplicated across the days and treatments.

**Figure 3:**
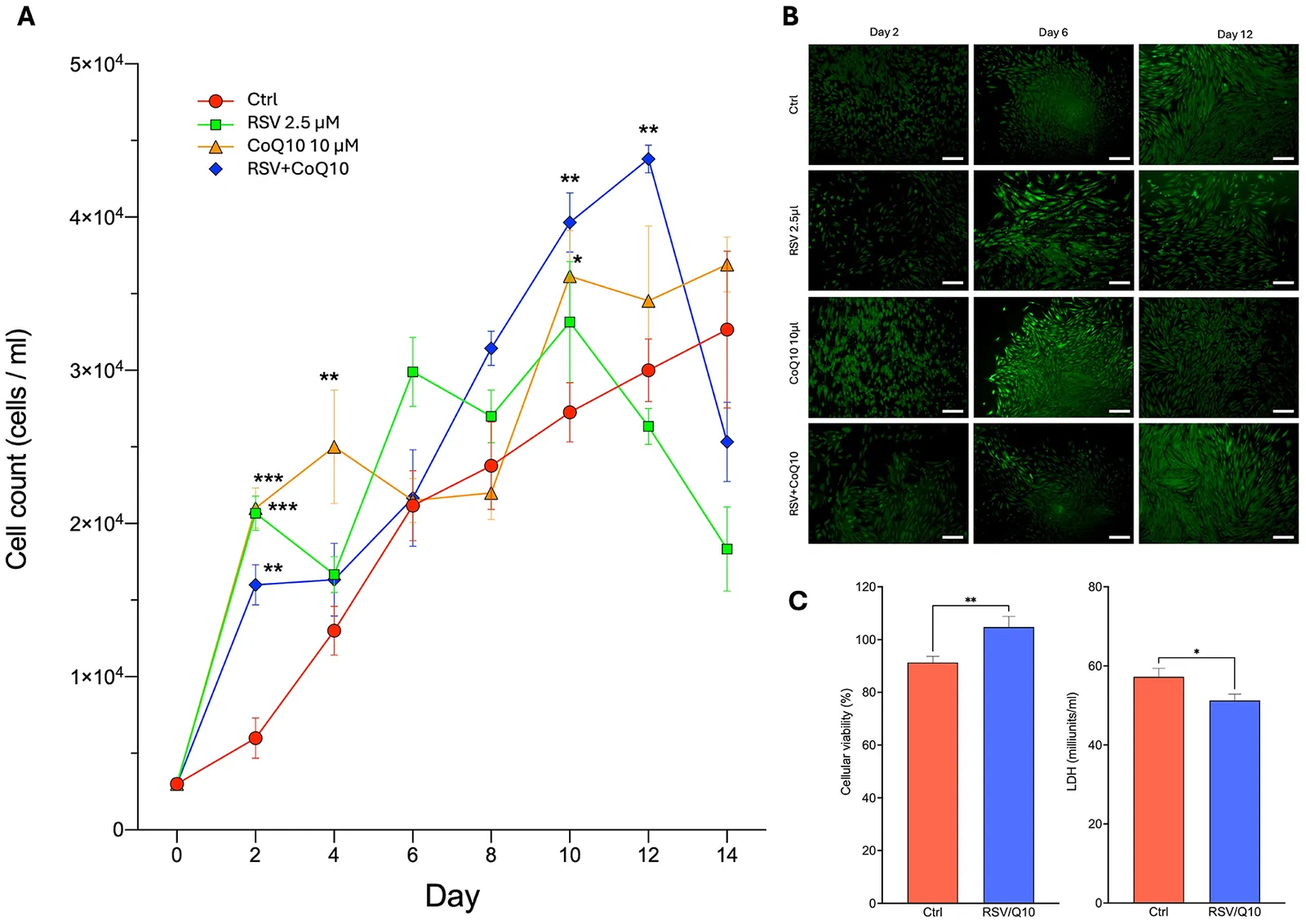
Effects of RSV + CoQ10 supplementation to human mesenchymal stem cells (hMSCs) on kinetic growth, cellular viability, and lactate dehydrogenase (LDH). (**A**) Applying RSV (2.5μM), CoQ10 (10μM), or combination to hMSCs increased the number of cells on day 2 vs. control. The RSV + CoQ10 combination significantly enhances the number of cells on days 10 and 12. (**B**) Representative pictures of confluency of hMSCs under fluorescence microscopy on three different days of kinetics. (**C**) On day 12, the application of RSV + CoQ10 increased cell viability. They reduced LDH levels compared to compare vs. control group. CoQ10, coenzyme Q10; RSV, resveratrol. *P < 0.05; **P < 0.01; ***P < 0.001. One-way ANOVA was followed by Dunnett’s test for kinetic growth curve and t-test for cell viability and LDH levels. Scale bar: 25μm.

We have replaced them with fluorescence photographs corresponding to the days and treatments.

The corrected Figure 3 appears below.

There was a mistake in the caption of Figure 3 as published.

The corrected Figure 3 caption appears below.

The original version of this article has been updated.

